# Autosomal recessive pathogenic *MSTO1* variants in hereditary optic atrophy

**DOI:** 10.15252/emmm.202216090

**Published:** 2023-07-11

**Authors:** Sylvie Gerber, Lola Lessard, Cécile Rouzier, Samira Ait‐el‐Mkadem Saadi, Roxana Ameli, Stéphane Thobois, Lucie Abouaf, Françoise Bouhour, Josseline Kaplan, Audrey Putoux, Antoine Pegat, Jean‐Michel Rozet

**Affiliations:** ^1^ IHU Imagine – Institut des Maladies Génétiques, Laboratoire de Génétique Ophtalmologique (LGO) Université Paris Descartes Paris France; ^2^ Service d'Electroneuromyographie et Pathologies Neuromusculaires Hôpital Neurologique Pierre Wertheimer, Hospices Civils de Lyon Bron France; ^3^ Service de Génétique Hôpital l'Archet 2, CHU de Nice Nice France; ^4^ Université Côte d'Azur, CHU, Inserm, CNRS, IRCAN Nice France; ^5^ Service de Neuroradiologie Hôpital Neurologique Pierre Wertheimer, Hospices Civils de Lyon Bron France; ^6^ Service de Neurologie C – Troubles du Mouvement et Pathologies Neuromusculaires Hôpital Neurologique Pierre Wertheimer, Hospices Civils de Lyon Bron France; ^7^ Cabinet d'Ophtalmologie des Tullistes Ecully France; ^8^ Unité de Génétique Clinique, Service de Génétique Centre Labellisé Anomalies du Développement, Hospices Civils de Lyon Bron France; ^9^ Centre de Recherche en Neurosciences de Lyon, Equipe GENDEV, INSERM U1028, UMR CNRS 5292 Université Claude Bernard Lyon 1 Bron France

## Abstract

Gerber *et al* report 2 autosomal recessive pathogenic Misato homolog 1 (MSTO1) variants causing hereditary optic atrophy and raise concerns about a previously identified dominant variant of MSTO1 by Gal *et al* (2017).

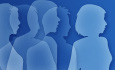

Misato homolog 1 (*MSTO1*) is a nuclear gene encoding a mitochondrial morphology‐regulating protein. Two forms of the *MSTO1* gene‐related disease have been reported: the recessive form was reported in more than 20 families segregating biallelic variants including large genomic deletions, changes affecting the initiation codon, nonsense variants, splice site variants, frameshift variants and missense variants (Human Gene Mutation database; https://www.hgmd.cf.ac.uk/ac/index.php), while the dominant form was reported in one family of four affected individuals (Val8Met, c.22G > A; Gal *et al*, 2017). The recessive form is characterized by an early‐onset proximal muscle weakness, elevated plasma creatine kinase (CK) levels, and additional symptoms, including nonprogressive cerebellar atrophy/ataxia, visual impairment, motor developmental delay, cognitive alteration, corticospinal tract involvement, and skeletal abnormalities (Donkervoort *et al*, 2019; Nasca *et al*, 2021), while the affected individuals from the dominant family suffered from cognitive and/or psychiatric disorders with distal muscle weakness and normal plasma CK levels.

Here, we report 2 biallelic *MSTO1* variants in a family presenting with early‐onset muscle weakness and adult‐onset optic neuropathy. Since the present study added *MSTO1* to the list of hereditary optic neuropathy (HON)‐causing genes (Gerber *et al*, 2021; Zeviani & Carelli, 2021), we further sequenced the *MSTO1* gene in a cohort of 49 patients with undiagnosed hereditary optic neuropathy.

The proband (III‐2) is a 63‐year‐old individual born to healthy unrelated parents of Jewish Ashkenazi origin (Fig 1A). He displayed childhood‐onset motor impairment, which remained relatively stable over time, and he subsequently developed progressive visual impairment by the age of 20 years. At 30 years of age, he presented with mild proximal limb weakness, and increased stretch reflex and positive Hoffman sign (Table 1). Neuro‐ophthalmic evaluation revealed a severe bilateral optic atrophy with no retinal involvement (Table 1). Further investigations showed moderately elevated CK levels (365 UI/l), myogenic features, and complex repetitive discharges (CRD) on electroneuromyography (ENMG), diffuse fat infiltration of the pelvic girdle and a peculiar pattern of relative sparing of several muscles on muscle MRI. Cardiac examination found no cardiomyopathy or heart conduction abnormalities (Fig 1B–D). Brain MRI revealed mild cerebellar atrophy. Next generation sequencing failed to detect pathogenic variants in the *OPA1* gene, the Leber hereditary optic neuropathy‐associated genes, nor in 384 nuclearly encoded mitochondrial genes. Two sibs of the proband (III‐1 and III‐3) also developed severe, adult‐onset optic neuropathy and complained of childhood‐onset muscle weakness. Whole exome sequencing of the trio, analyzed according to both dominant and recessive models, identified 2 allelic variants in the *MSTO1* gene: a missense variant NM_018116: c.65C > A (p.Ala22Glu) absent from public databases, and an ultra‐rare splicing variant c.220 + 5G > C (rs1187504822; minor allele frequency, MAF: 0.0032%) predicted to disrupt the splice donor site of intron 2 (Fig 1E). Both variants were predicted as “pathogenic” according to the American College of Medical Genetic (ACMG) classification (Richard *et al*, 2015). Biallelism was confirmed by Sanger sequencing‐based segregation analysis (Fig 1A), and cDNA analysis from patient's cultured fibroblasts showed apparent homozygosity of the c.65C > A variant (Fig 1E and F), suggesting instability or degradation of the mRNA transcribed from the allele carrying the c.220 + 5G > C substitution.

**Figure 1:**
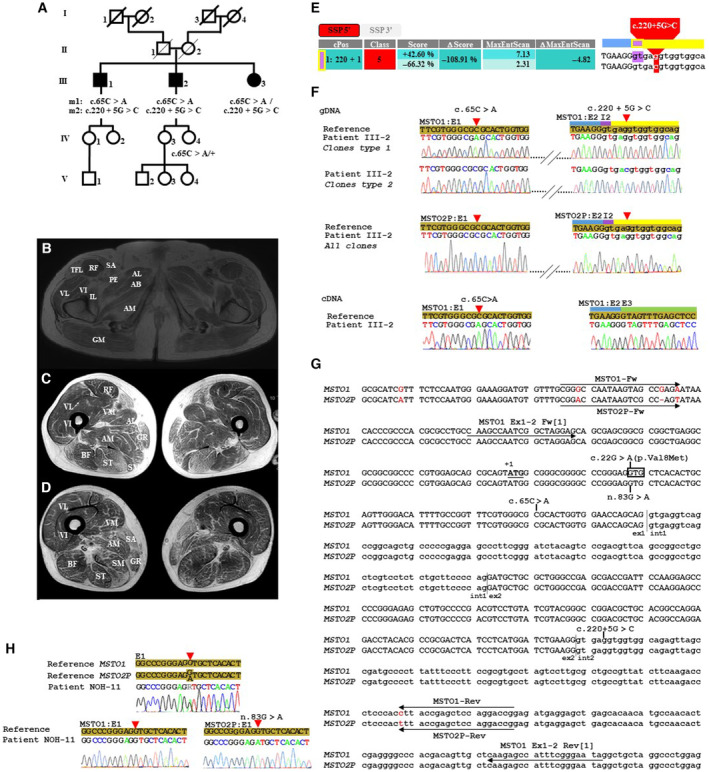
Family, muscular findings, *MSTO1* and *MSTO2P* sequences alignment and variants analysis (A) Pedigree of the family and segregation analysis of *MSTO1* c.65C > A and c.220 + 5G > C variations. The proband (III‐2) and his 2 siblings (III‐1 and III‐3) developed early‐onset myopathy and severe optic neuropathy. Segregation analysis of *MSTO1* variants using the *MSTO1* Fw/*MSTO1* Rev primer pair (specific to *MSTO1*; see panel G) shows heterozygosity for the c.65C > A substitution in unaffected subject IV‐4, demonstrating biallelism of the c.65C > A and c.220 + 5G > C variations in the affected trio III‐1, ‐2, and ‐3. (B–D) Muscle MRI. Axial T1‐weighted imaging on pelvis (B), upper thigh (C), and lower thigh (D) showing major fat infiltration of the thigh muscles. AB, adductor brevis muscle; AL, adductor longus muscle; AM, adductor magnus muscle; BF, biceps femoris muscle; GM, gluteus maximus muscle; GR, gracilis muscle; IL, ilio‐psoas muscle; PE, pectineus muscle; RF, rectus femoris muscle; SA, sartorius muscle; SM, semimembranosus muscle; ST, semitendinosus muscle; TFL, tensor fasciae latae; VL, vastus lateralis muscle; VM, vastus medialis muscle; VI, vastus intermedius muscle. (E) 5′ Donor Splice Site Prediction (SSP) by varSEAK SSP tool. The varSEAK Online splice site prediction tool was used to analyze the c.220 + 5G > C variant. The table shows a strong decrease in the score for authentic Splice Site (Pos 220 + 1, highlighted in purple in the table as well as on the graph on the right). The score corresponds to the likelihood for the variant to be functional (positive values) or not functional (negative values). The score column corresponds to the score of the splice site on the reference sequence (upper value) and on the variant sequence (lower value). The ΔScore corresponds to the difference between the two scores. There was a strong decrease in the score and ΔScore for the 5′ authentic Splice Site, thus predicting the loss of function and exon skipping of the variant. The overall prediction is given as a splice site prediction class ranging from 1 (No splicing effect) to 5 (Splicing effect). The c.220 + 5G > C variant was classified as affecting splicing (class 5). No cryptic splice sites were predicted to be activated. The MaxEntScan scoring algorithm also found a decrease in the score and negative values for the ΔMaxEntScan, thus predicting a deleterious effect. The sequence graph (on the right) depicts the reference and variant sequence and the 5′ authentic Splice Site. The variant is highlighted in red, the HGVS nomenclature is given above. (F) Sanger sequencing of the identified variants in the *MSTO1* gene and RT–PCR analysis. Upper panel: Representative sequence chromatograms of PCR products generated using the M13F/M13R primers of the pCR™II‐TOPO^R^ Vector carrying the *MSTO1* c.65A (mutant)—c.220 + 5G (wildtype) allele (Clones type 1) or the *MSTO1* c.65C (wildtype)—c.220 + 5C (mutant) allele (Clones type 2), amplified from whole blood genomic DNA (gDNA) using the MSTO1 Fw/MSTO1 Rev primers (specific to *MSTO1*; see panel G). Middle panel: Representative sequence chromatograms of pCR™II‐TOPO^R^ Vector clones carrying *MSTO2P* alleles amplified from whole blood genomic DNA (gDNA) using MSTO2P Fw/MSTO2P Rev primer pair (specific to *MSTO2P*; see panel G) and showing wildtype exon 1 and exon2‐intron 2 junction sequences. Lower panel: Representative sequence chromatograms of reverse‐transcribed mRNA isolated from patient‐cultured fibroblasts, showing apparent homozygosity of the *MSTO1*‐mutated allele (A) at position c.65 (on the left). On the right, the chromatogram shows the patient sequence at the exon 2/exon 3 junction of *MSTO1* cDNA. E1, E2, E3, and I2 denotes exons 1, 2, and 3 and intron 2, respectively. The bars above the chromatograms show the boundaries of exon 2 (blue), intron 2 (yellow), and/or exon 3 (green) and the authentic consensus splice donor site (purple). (G) Pairwise alignment of *MSTO1* and *MSTOP2* and position of primer pairs reported in [1] and specific to *MSTO1* and *MSTOP2* [this study]. A noncoding RNA *MSTO1* pseudogene, misato homolog 2 pseudogene (*MSTO2P*), is predicted 133 kb downstream of *MSTO1* (hg19_chr1:155579961–155584758 and chr1:155715559–155718322, respectively). Pairwise alignment (https://www.ebi.ac.uk/Tools/psa/lalign/) of the proximal 5′ region, exon 1, intron 1, and part of exon 2 of *MSTO1* and *MSTO2P* shows 99.8% nucleotide identity; the few variable positions are shown in red. *MSTO1* Ex1‐2 Fw and *MSTO1* Ex1‐2 Rev primers reported in [1] and MSTO1 Fw/MSTO1 Rev and MSTO2P Fw/MSTO2P Rev primer pairs specific to MSTO1 and MSTO2P, respectively, are shown. The +1 indicates the position of the adenine of the initiation codon (ATG is underlined). The codon involving the *MSTO1* c.22 nucleotide is squared. The *MSTO1* c.65C > A and c.220 + 5G > C are shown. Exonic and intronic sequences are written in lower and uppercase letters, respectively. (H) Sanger sequencing analysis of the *MSTO2P* n.83G > A substitution using primers reported in [1] and primers specific to *MSTO1* and *MSTO2P*, respectively. Representative Sanger sequencing electrophoregrams in one of the HON cases carrying the *MSTO2P* n.83G > A substitution (patient NOH‐11). Sequences were obtained from PCR products obtained using MSTO1 Ex1‐2 Fw/MSTO1 Ex1‐2 Rev [1] primers (upper panel) and from PCR products amplified using the M13F/M13R primers of the pCR™II‐TOPO^R^ Vector carrying *MSTO1* or *MSTO2P* alleles amplified using the MSTO1 Fw / MSTO1 Rev (lower‐left panel) and MSTO2P Fw/MSTO2P Rev (lower right panel) primers, respectively.

**Table 1:** Genetic and Clinical features of the patients from the family with *MSTO1* mutations

Patients	(III‐2)	(III‐1)	(III‐3)
***MSTO1* mutation**			
Missense variant	c.65C > A	c.65C > A	c.65C > A
Frameshift variant	c.220 + 5G > C	c.220 + 5G > C	c.220 + 5G > C
**Muscle**	**Early‐onset proximal myopathy, relatively stable course over the years**		
Clinical examination	Unlimited walking distance at age 63, Waddling gait, Impossible heel‐to‐toe walking, Gower's sign, proximal limb‐girdle weakness (Medical Research Council 4/5)	Not examined. History of lower limb muscular weakness complain	Not examined. History of lower limb muscular weakness complain
CK level	365 IU/l	/	/
Lactate/pyruvate	Normal	Normal	/
EDX	Myogenic features, complex repetitive discharges, mild sensitive axonal polyneuropathy of the lower limbs	/	/
Muscle MRI	Gluteus maximus, the tensor fasciae latae, the sartorius, the adductor magnus, and the semitendinosus muscles = most severely affected muscles, relative sparing of the ilio‐psoas, the quadricipital, the adductor longus and brevis, and the biceps femoris muscles. Unremarkable scapular muscles No pathological enhancement after gadolinium infusion or T2 hyperintensities	/	/
Muscle biopsy	Not performed	/	/
**Corticospinal tract**			
Clinical examination	Increased stretch reflex, positive Hoffman sign, without Babinski sign	/	/
**Cerebellum**			
Clinical examination	No ataxia, no dysarthria	/	/
Brain MRI	Cerebellar atrophy	/	/
**Eye**	**Secondary onset, progressive visual impairment with age, severe optic neuropathy in adult life**		
Clinical examination	History of visual decline around 20 years. Optic neuropathy diagnosed at age 30 with severe bilateral visual acuity loss (20/320 Snellen equivalent). Finger counting was possible at 1 m at age 63	History of visual decline around 25 years. Severe bilateral visual acuity loss at 54 years (20/100 Snellen equivalent)	History of visual decline around 48–49 years. Optic neuropathy diagnosed at age 53 with optic nerve pallor, severe bilateral visual acuity loss (20/320 Snellen equivalent), central (RE), and caeco‐central (LE) scotoma
Visual evoked potential	Impaired visual evoked potential	/	/
Optical coherence tomography	Severe decrease of retinal nerve fiber layer thickness and ganglion cell layer volume (SD–OCT, Spectralis, Heidelberg, Germany)	/	/
Electroretinogram	Normal full‐field and macular component of pattern electroretinogram (Metrovision, Pérenchies, France)	/	/
Genetic testing for HON	Negative for *OPA1* and LHON‐associated genes, negative for 384 nuclear mitochondrial genes (NGS)	/	/
**Additional features**			
Cognitive and/or psychiatric symptoms	None	/	/
Skeletal abnormalities	None	/	/

While retinitis pigmentosa has previously been reported in *MSTO1* gene‐related disease, this study first reports on severe, bilateral optic neuropathy in the disease and suggests that *MSTO1* should be added to the list of hereditary optic neuropathy (HON)‐causing genes (Gerber *et al*, 2021; Zeviani & Carelli, 2021) especially when associated with muscular involvement mimicking congenital muscle dystrophy.

Interestingly, we retrospectively screened *MSTO1* in 49 patients with unexplained HON and we detected the previously reported dominant variant (c.22G > A, p.Val8Met; Gal *et al*, 2017) in 16/49 patients (32.6%), questioning the relevance of this variant. We thus performed further analyses to investigate this high frequency; pairwise alignment of the *MSTO1* RNA with the non‐coding RNA transcribed from the *MSTO1* pseudogene (*MSTO2P*, located 133 kb downstream) revealed a perfect match of *MSTO1* Ex1‐2 Fw and Rev primers (Gal *et al*, 2017) and a unique mismatch in the 563 bp amplified region (Fig 1G), showing a lack of specificity of these primers. In this region, the *MSTO1* nucleotide involved in the reportedly dominant c.22G > A substitution aligns to the *MSTO2P* r.83 position (Fig 1G) where a frequent G > A polymorphism is described (NR_024117.2: n.83G > A, rs11264409, minor allele frequency 32.6%). We amplified *MSTO2P* and *MSTO1* individually using specific primers (Fig 1G) and cloned the products in the pCR™II‐TOPO^R^ Vector. Sanger sequencing of the constructs using a vector‐specific primer showed the presence of the *MSTO2P* n.83G > A variant in the 16 patients of the HON cohort and the proband (III‐2), and confirmed the location of c.65C > A and c.220 + 5G > C substitutions on each of the two *MSTO1* alleles in the proband (III‐2), respectively (Fig 1H). Knowing the elevated frequency of the n.83G > A polymorphism, it is intriguing that in the dominant family, the authors reported dramatic silencing or decay of *MSTO1* mRNA while there was a notable detection of a G > A change at the RNA level (Gal *et al*, 2017). Whether these observations describe the presence of the G > A substitution on *MSTO2P* instead of *MSTO1* needs consideration before the dominant *MSTO1* c.22G > A substitution referred to in the CLINVAR catalog (RCV000505814.2) is definitely validated.

To conclude, *MSTO1* should be considered in patients with optic atrophy and muscular involvement, especially if mimicking congenital muscle dystrophy, and geneticists/clinicians should consider the presence of a highly homologous pseudogene in *MSTO1* diagnosis, questioning the reality of dominant forms of the disease.

## Methods

Family 1 (Fig 1A) was selected for the study due to negatively targeted exome sequencing‐based molecular diagnosis of mitochondrial DNA and a panel of 384 genes, including genes known to be associated with mitochondrial disorders and genes for complex I subunits and assembly factors. The *Comité de Protection des Personnes Ile–de–France II* Institutional Review Board approved all aspects of the study herein described (CPP:2015‐03‐03/DC2014‐2272). Informed consent was obtained from all subjects involved in the study, and experiments conformed to principles set out in the WMA Declaration of Helsinki and Human Services Belmont Report. The proband gave his permission to publish the present work.

### Whole exome sequencing analysis

Genomic DNA was extracted from whole blood using standard protocols. The DNA (1 μg) of the proband and his affected brother and sister were sequenced using the HiSeq 2000 (Illumina) at the Genomic Core Facility of Imagine (Paris). Libraries were prepared using the SureSelect Human All Exon Kit v3 (Agilent Technologies) according to the manufacturer's protocols and run in pair‐ended mode (2 × 75). Image analysis and base calling were performed with Real Time Analysis (RTA) Pipeline version 1.9 with default parameters (Illumina). Sequences were aligned to the human genome reference sequence (UCSC Genome Browser hg19 assembly), and single nucleotide polymorphisms were called on the basis of allele calls and read depth with the use of the Consensus Assessment of Sequence and Variation pipeline (v.1.8, Illumina). Genetic variation annotation was performed by an in‐house pipeline. Only the variants whose positions were covered ≥ 10× were further considered. We selected compound heterozygous variants with a minor allele frequency < 1% (recessive model) and heterozygous variants with a minor allele frequency < 0.1% (dominant model) shared by the three affected members. The pathogenicity of the selected variants was assessed using the Alamut Mutation Interpretation Software (http://www.interactive-biosoftware.com), a decision support system for mutation interpretation based on Align DGVD, MutationTaster, PolyPhen‐2, SIFT, SpliceSiteFinder‐like, MaxEntScan, NNSPLICE, GeneSplicer, Human Splicing Finder, ESEfinder, and RESCUE‐ESE. The effect of the c.220 + 5G > C substitution on splicing was further evaluated using the varSEAK online splice site prediction tool (https://varseak.bio/) and MaxEntScan, respectively.

### PCR amplification of MSTO1 and MSTO2P, respectively

PCRs (10 μl) were performed on an Applied Biosystems^®^ 2720 thermal cycler (Thermo Fisher, Villebon‐sur‐Yvette, France) in 1X Green Go Taq Flexi Buffer (Promega, Charbonnières‐les‐Bains, France) containing genomic DNA (20 ng; extracted from circulating blood white cells by salt precipitation and ethanol recovery), primer pairs (0.2 μM each; MSTO1 Fw: 5′‐CGGGCCAATAAGTAGCCGAGA‐3′ and MSTO1 Rev: 5′‐CGGTCCTGGAGCTCGGTAAG‐3′ primer and MSTO2P Fw: 5′‐CGGACCAATAAGTCGCCAGT and MSTO2P Rev: 5′‐CGGTCCTGGAGCTCGGTAAA‐3′ for *MSTO1* and *MSTO2P*, respectively), MgCl_2_ (1.5 mM, Promega), dNTP (0.04 mM, Promega), and Go Taq G2 Hot Start Polymerase (0.015 units; Promega). A touchdown PCR program was used to increase the specificity of annealing, which consisted of initial denaturation at 98°C for 3 min; 8 cycles of denaturation at 98°C for 20 s, annealing from 66°C to 58°C for 15 s (−1°C each cycle), extension at 72°C for 30 s; 27 cycles of denaturation at 98°C for 20 s, annealing at 58°C for 15 s and extension at 72°C for 30 s, and a final extension at 72°C for 1 min.

### Cloning and Sanger sequencing of MSTO1 or MSTO2P‐containing colonies

*MSTO1* or *MSTO2P* amplifications (2 μl) were verified on a 2% agarose gel and cloned (2 μl) in the pCR™ II‐TOPO^R^ Vector using the TOPO™ TA Cloning™ Kit, dual promoter, with pCR™II‐TOPO™ Vector and One Shot™ Mach1™ T1 phage‐resistant chemically competent *Escherichia coli* according to the manufacturer's protocol Invitrogen, Life Technologies, St Aubin, France.

*MSTO1* and *MSTO2P* sequences were amplified by PCR (10 μl) directly from bacteria colonies in 1X Green Go Taq Flexi Buffer (Promega) containing the vector‐specific M13F and M13R primers (0.2 μM each), MgCl2 (1.5 mM, Promega), dNTP (0.04 mM, Promega), and Go Taq G2 Hot Start Polymerase (0.015 units; Promega). The PCR program consisted of an initial denaturation at 98°C for 3 min, 30 cycles of denaturation at 98°C for 20 s, annealing at 58°C for 20 s, extension at 72°C for 30 s, and a final extension at 72°C for 1 min.

Amplifications (2 μl) were verified on a 2% agarose gel and purified (8 μl) from single‐strand DNA by adding exonuclease I (4 units; Thermo Fisher) and FastAP thermosensitive alkaline phosphatase (0.4 unit; Thermo Fisher) for 10 min at 37°C. The reactions were arrested by heating the samples at 85°C for 10 min.

Sequencing reactions (10 μl) were performed on purified PCR products (2.5 μl) using the vector‐specific M13F primer and the Applied Biosystems^®^ BigDye Terminator v3.1 Cycle Sequencing Kit on an Applied Biosystems^®^ 2720 thermal cycler with the following cycling conditions: an initial denaturation at 98°C for 3 min, 35 cycles of denaturation at 98°C for 20 s, annealing at 58°C for 20 s, extension at 60°C for 40, and a final extension at 60°C for 5 min.

Sequencing products in nuclease‐free water (10 μl/10 μl) were purified from oligonucleotides and unincorporated ddNTPs and dNTPs by spin column size exclusion chromatography using Sephadex G‐50 (GE Healthcare) on 96‐well multiscreen 0.45 μm Durapore^®^ Membrane plates (Merck Millipore; Guyancourt, France) for 4 min at 18 *g*. Purified products were analyzed on an Applied Biosystems^®^ 3500XL genetic analyzer.

### Cell culture

A skin biopsy was obtained from the proband III‐2 and a control (Fig 1). Primary fibroblasts were isolated by selective trypsinization and proliferated at 37°C, 5% CO_2_ in Opti‐MEM Glutamax I medium (Invitrogen) supplemented with 10% fetal bovine serum (Invitrogen), 1% ultroser G substitute serum (Pall, Saint‐Germain‐en‐Laye, France), and 1% streptomycin/penicillin (Invitrogen).

### RNA preparation, cDNA synthesis, and RT–PCR analysis of MSTO1

Total RNA was prepared from fibroblasts using the RNeasy Mini Kit (QIAGEN, Les Ulysses, France) in accordance with the manufacturer's protocol. The samples were DNase‐treated with the RNase‐free DNase Set (QIAGEN). The concentration was assessed using the NanoDrop spectrophotometer (ThermoFisher) before storage at −80°C. First‐strand cDNA synthesis (20 μl) was performed from total RNA (500 ng) using the Verso cDNA Kit (ThermoFisher Scientific) with random hexamer:anchored oligo (dT) primers in a 3:1 (vol:vol) ratio, in accordance with the manufacturer's instructions. A non‐RT reaction (without an enzyme) for one sample was prepared as a control. *MSTO*1cDNA (2 μl) was amplified by PCR (10 μl) using the MSTO1 Fw and MSTO1 Rev primers specific to *MSTO1* (Fig 1G) and Sanger sequenced using the MSTO1 Fw or MSTO1 Rev primers individually, following the protocol described in “Cloning and Sanger sequencing of MSTO1 and MSTO2P clones.”

## Author contributions

**Sylvie Gerber:** Data curation; formal analysis; validation; investigation; visualization; methodology; writing – review and editing. **Lola Lessard:** Resources; data curation; validation; investigation; writing – original draft. **Cécile Rouzier:** Validation; investigation; visualization; methodology; writing – review and editing. **Samira Ait‐el‐Mkadem Saadi:** Investigation; writing – review and editing. **Roxana Ameli:** Investigation; writing – review and editing. **Stephane Thobois:** Investigation; writing – review and editing. **Lucie Abouaf:** Investigation; writing – review and editing. **Francoise Bouhour:** Investigation; writing – review and editing. **Josseline Kaplan:** Resources; funding acquisition; writing – review and editing. **Audrey Putoux:** Resources; writing – review and editing. **Antoine Pegat:** Conceptualization; supervision; validation; visualization; writing – review and editing. **Jean‐Michel Rozet:** Conceptualization; supervision; funding acquisition; visualization; project administration; writing – review and editing.

In addition to the CRediT author contributions listed above, the contributions in detail are:

Sylvie Gerber involved in investigation, methodology, formal analysis, data curation, validation, visualization (Genetic and Biomolecular studies), and writing—review and editing. Lola Lessard involved in resources (Provision of Clinical and Genetic data and Patient samples), investigation, validation, data curation (neuromuscular assessment and interpretation), and writing—original draft. Cécile Rouzier involved in investigation, methodology, validation, visualization (Biomolecular studies), and writing—review and editing. Samira Ait‐el‐Mkadem Saadi involved in investigation (Biomolecular studies), and writing—review and editing. Roxana Ameli involved in investigation (radiological data interpretation), and writing—review and editing. Stephane Thobois involved in investigation (neuromuscular assessment and interpretation), and writing—review and editing. Lucie Abouaf involved in investigation (ophthalmological data interpretation), and writing—review and editing. Françoise Bouhour involved in investigation (neuromuscular assessment), and writing—review and editing. Josseline Kaplan involved in resources (Provision of Clinical and Genetic data and Patient samples), funding acquisition, and writing—review and editing. Audrey Putoux involved in resources (Provision of Clinical and Genetic data and Patient samples), and writing—review and editing. Antoine Pégat involved in conceptualization, supervision, validation, visualization, and writing—review and editing. Jean‐Michel Rozet involved in conceptualization, supervision, project administration, funding acquisition, visualization, and writing—review and editing.

## Supporting information



## Data Availability

Whole exome sequencing .bam files are deposited in the access‐controlled *Interactive Polyweb interface* (https://polyweb.fr/) of the Institute of Genetic Diseases, *Imagine* – University Paris Descartes. They can be accessed upon a duly substantiated request. The *MSTO1* c.65C > A and c.220 + 5G > C variants have been deposited in the CLINVAR database with the accession references SCV002526430 and SCV002526431, respectively.
