# Supplementary material for: Autosomal recessive pathogenic MSTO1 variants in hereditary optic atrophy
Source: EMBO Mol Med. 2023 Jul 11;15(8):e16090. doi: 10.15252/emmm.202216090 (PMC10405053; doi:10.15252/emmm.202216090)
Supplement: Supplementary file 1 [file EMMM-15-e16090-s001.pdf]

EMBO Molecular Medicine

Autosomal recessive pathogenic MSTO1 variants in hereditary optic atrophy

Sylvie Gerber, Lola Lessard, Cecile Rouzier, Samira Saadi, Roxana Amelie, Stephane Thobois, Lucie Abouaf, Francoise Bouhour, Josseline Kaplan, Audrey Putoux, Antoine Pegat, and Jean-Michel Rozet
DOI: 10.15252/emmm.202216090

Corresponding author(s): Jean-Michel Rozet (jean-michel.rozet@inserm.fr) , Antoine Pegat (antoine.pegat@chu-lyon.fr)

Review Timeline:

Submission Date:	4th Apr 22
Editorial Decision:	10th Jun 22
Revision Received:	16th Jun 22
Accepted:	18th Jan 23

Editor: Jingyi Hou

Transaction Report:

(Note: With the exception of the correction of typographical or spelling errors that could be a source of ambiguity, letters and reports are not edited. Depending on transfer agreements, referee reports obtained elsewhere may or may not be included in this compilation. Referee reports are anonymous unless the Referee chooses to sign their reports.)

Dear Dr. Rozet,

Thank you again for submitting your Correspondence article to EMBO Molecular Medicine. We have now heard back from two referees. As you will see below, the referees think the analyses and findings are relevant and important.

As I mentioned before, the authors of the related EMBO Mol Med paper (Gal et al., 2017) were given an opportunity to prepare a reply to your Correspondence, which has now also been reviewed by the same referees. We are currently in the process of contacting the authors to discuss how to proceed.

On a more editorial level, please do the following:

1. Figures: Please upload individual, high-resolution figure file. Figure legend should remain at the end of the main manuscript text. For more information on figure presentation please check "Author Guidelines".
https://www.embopress.org/page/journal/17574684/authorguide#datapresentationformat

2. In the main manuscript file, please do the following:
- remove " not shown". As per our guidelines, on "Unpublished Data" the journal does not permit citation of "Data not shown". All data referred to in the paper should be displayed in the main or Expanded View figures.
- Add "Disclosure Statement & Competing Interests". We updated our journal's competing interests policy in January 2022 and request authors to consider both actual and perceived competing interests. Please review the policy https://www.embopress.org/competing-interests and update your competing interests if necessary.

3. Please provide 2 sentences that summarise the main message of the article.

4. The current Table is too large. Please compress the current table into a portrait-oriented A4 page and provide them in a .doc format separately. Please make sure that the width of the tables does not exceed an A4 page.

5. Funding: Please make sure that information about all sources of funding are complete in both our submission system and in the manuscript.

As part of the EMBO Publications transparent editorial process initiative (see our Editorial at http://embomolmed.embopress.org/content/2/9/329), EMBO Molecular Medicine will publish online a Review Process File (RPF) to accompany accepted manuscripts. This file will be published in conjunction with your paper and will include the anonymous referee reports and all pertinent correspondence relating to the manuscript. Let us know if you disagree with the publication of the RPF and as here, if you want to remove or not any figures from it prior to publication.

You can submit your revised files by logging onto our online manuscript tracking system or simply follow this link:

https://embomolmed.msubmit.net/cgi-bin/main.plex

Kind regards,
Jingyi

***** Reviewer's comments *****

Referee #1 (Novelty/Model system Comments for Author):

This correspondence reports two new recessive mutations in MSTO1 that extend the clinical phenotype associated with loss of function. Importantly it also points out that a dominant (missense)mutation (n.22G>A)in MSTO1, reported in a single pedigree, may in fact be in the pseduogene MSTO2P. The single nucleotide substitution (n.83G>A)in MSTO2P which aligns with the 22G>A mutation has a minor allele frequency of more than 0.3, thus calling into question its pathogenecity.

Referee #1 (Remarks for Author):

No specific remarks. The manuscript is clear and well written and likely clears up a misconception in a previous report of dominant inheritance of MSTO1.

Referee #2 (Remarks for Author):

The authors report two biallelic pathogenic MTSO1 variants in a family presenting with early-onset muscle weakness and adult-onset optic neuropathy. One of the variants NM_018116: c.65C>A (p.Ala22Glu) is a significant change that is likely pathogenic. While the other variant is predicted to affect splicing. Analysis of the cDNA could only detect the c.C>A variant, suggesting mRNA mediated decay of the pathogenic splicing variant. The data as a clinical report appears sound, indicating the existence of additional pathogenic MTSO1 variants associated with autosomal recessive disorders.

During retrospective screening with patients with unexplained hereditary optic neuropathy, the authors identify the Val8Met variant that was previously associated with an autosomal dominant disorder by Gal et al. 2017. Further, the authors propose that this variant in fact originates in a nearby pseudogene MSTOP2. As a consequence, the clinical relevance of the Val8Met as pathogenic is questioned by the authors, who urge a re-evaluation before inclusion into the ClinVar archive. The rationale and evidence provided Gerber et al to question the pathogenicity of the Val8Met variant is warranted.

Point by point response to the referees

Referee #1
No specific remarks. The manuscript is clear and well written and likely clears up a misconception in a previous report of dominant inheritance of MSTO1.

Referee #2
The rationale and evidence provided Gerber et al to question the pathogenicity of the Val8Met variant is warranted.

We are very grateful to the two referees for dedicating time to evaluate of work and their very positive evaluation.
We would like to draw their attention to the changes made in the manuscript and Figure to fit the EMBO Mol Med Editorial requirements:

-Figure 1 has been has been reviewed to show data confirming the location of the c.65C>A and c.220+5G>C substitutions on *MSTO1* alleles: The panel F now includes a middle panel which displays sequence chromatograms showing that the proband carries wildtype *MSTO2P* exon 1 and exon2-intron 2 junction sequences. The *MSTO1* the c.65C>A and c.220+5G>C substitutions have been added on Fig. 1G. A high resolution version of the figure has been provided.

-Two sentences have been provided that summarise the main message of the article: “Here, we report novel *MSTO1* pathogenic variants in a previously unreported association of myopathy, cerebellar atrophy and corticospinal involvement with severe visual loss due to bilateral optic atrophy, widening the spectrum of MSTO1-associated diseases and optic neuropathy-causing genes. Also, we shed light on a highly homologous pseudogene MSTO2P, the study of which questions the existence of a dominant *MSTO1*-associated disease.”

-The Table has been reformatted to fit on an A4 page.

-We have included the ACMG classification of the two novel *MSTO1* variants identified in the proband and affected brother and sister. The supporting reference has been listed in the ‘Reference’ section.

-The funding sources, ethical aspects and reference to core facility services have been added in the ‘Acknowledgement section’.

-Authors contributions have been reviewed to fit the CRediT recommendations.

-A ‘Data availability’ section has been added which include information regarding accessibility of WES .Bam files and will provide the CLINVAR references of the novel MSTO1 variants (data submitted to CLINVAR on 14/06/2022; waiting for validation).

Yours sincerely,

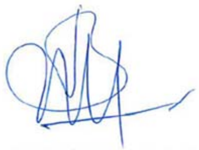

Jean-Michel Rozet, PhD
Laboratory Genetics in Ophthalmology
INSERM UMR1163
Institute of genetic diseases, Imagine

18th Jan 2023

Dear Dr. Rozet,

Please find enclosed the final reports on your manuscript. We are pleased to inform you that your manuscript is accepted for publication.

If you want to receive an e-mail alert regarding its publication as well as other EMBO Mol Med content, register here: embopress.org/alertsfeeds

Please read below for additional IMPORTANT information regarding your article, its publication and the production process.

Congratulations on your interesting work,
Jingyi

Jingyi Hou
Editor
EMBO Molecular Medicine

Follow us on Twitter @EmboMolMed

***** Reviewer's comments *****

Referee #1 (Novelty/Model system Comments for Author):

The authors have identified novel biallelic mutations in MSTO1, and quite convincingly shown that a previously reported dominant mutation, with a very high population allele frequency, is actually present in a pseudogene, calling into question the relevance to disease.

Referee #1 (Remarks for Author):

Fine for EMBO Mol Med

Referee #2 (Remarks for Author):

The authors have adequately addressed all concerns raised in the review.

EMBO Press Author Checklist

Corresponding Author Name: Jean-Michel ROZET	
Journal Submitted to: EMBO Molecular Medecine	
Manuscript Number: EMM-2022-16090	

USEFUL LINKS FOR COMPLETING THIS FORM
The EMBO Journal - Author Guidelines
EMBO Reports - Author Guidelines
Molecular Systems Biology - Author Guidelines
EMBO Molecular Medicine - Author Guidelines

Reporting Checklist for Life Science Articles (updated January 2022)
This checklist is adapted from Materials Design Analysis Reporting (MDAR) Checklist for Authors. MDAR establishes a minimum set of requirements in transparent reporting in the life sciences (see Statement of Task: 10.31222/osf.io/9sm4x). Please follow the journal's guidelines in preparing your manuscript.
Please note that a copy of this checklist will be published alongside your article.

Abridged guidelines for figures
1. Data
The data shown in figures should satisfy the following conditions:
- the data were obtained and processed according to the field's best practice and are presented to reflect the results of the experiments in an accurate and unbiased manner.
- ideally, figure panels should include only measurements that are directly comparable to each other and obtained with the same assay.
- plots include clearly labeled error bars for independent experiments and sample sizes. Unless justified, error bars should not be shown for technical replicates.
- if n<5, the individual data points from each experiment should be plotted. Any statistical test employed should be justified.
- Source Data should be included to report the data underlying figures according to the guidelines set out in the authorship guidelines on Data Presentation.

2. Captions
Each figure caption should contain the following information, for each panel where they are relevant:
- a specification of the experimental system investigated (eg cell line, species name).
- the assay(s) and method(s) used to carry out the reported observations and measurements.
- an explicit mention of the biological and chemical entity(ies) that are being measured.
- an explicit mention of the biological and chemical entity(ies) that are altered/varied/perturbed in a controlled manner.
- the exact sample size (n) for each experimental group/condition, given as a number, not a range;
- a description of the sample collection allowing the reader to understand whether the samples represent technical or biological replicates (including how many animals, litters, cultures, etc.).
- a statement of how many times the experiment shown was independently replicated in the laboratory.
- definitions of statistical methods and measures:

- common tests, such as t-test (please specify whether paired vs. unpaired), simple χ2 tests, Wilcoxon and Mann-Whitney tests, can be unambiguously identified by name only, but more complex techniques should be described in the methods section;
- are tests one-sided or two-sided?
- are there adjustments for multiple comparisons?
- exact statistical test results, e.g., P values = x but not P values < x;
- definition of 'center values' as median or average;
- definition of error bars as s.d. or s.e.m.

Please complete ALL of the questions below.
Select "Not Applicable" only when the requested information is not relevant for your study.

Materials

Newly Created Materials	Information included in the manuscript?	In which section is the information available? (Reagents and Tools Table, Materials and Methods, Figures, Data Availability Section)
New materials and reagents need to be available; do any restrictions apply?	Not Applicable	

Antibodies	Information included in the manuscript?	In which section is the information available? (Reagents and Tools Table, Materials and Methods, Figures, Data Availability Section)
For **antibodies** provide the following information:<br>- Commercial antibodies: RRID (if possible) or supplier name, catalogue number and or/clone number<br>- Non-commercial: RRID or citation	Not Applicable	

DNA and RNA sequences	Information included in the manuscript?	In which section is the information available? (Reagents and Tools Table, Materials and Methods, Figures, Data Availability Section)
**Short novel DNA or RNA including primers, probes**: provide the sequences.	Yes	Primer sequences_Figure and Legend

Cell materials	Information included in the manuscript?	In which section is the information available? (Reagents and Tools Table, Materials and Methods, Figures, Data Availability Section)
**Cell lines:** Provide species information, strain. Provide accession number in repository **OR** supplier name, catalog number, clone number, and/**OR** RRID.	Not Applicable	
**Primary cultures:** Provide species, strain, sex of origin, genetic modification status.	Yes	Proband-derived primary fibroblasts_mstxt
Report if the cell lines were recently **authenticated** (e.g., by STR profiling) and tested for mycoplasma contamination.	Not Applicable	

Experimental animals	Information included in the manuscript?	In which section is the information available? (Reagents and Tools Table, Materials and Methods, Figures, Data Availability Section)
**Laboratory animals or Model organisms:** Provide species, strain, sex, age, genetic modification status. Provide accession number in repository **OR** supplier name, catalog number, clone number, **OR** RRID.	Not Applicable	
**Animal observed in or captured from the field:** Provide species, sex, and age where possible.	Not Applicable	
Please detail **housing and husbandry conditions**.	Not Applicable	

Plants and microbes	Information included in the manuscript?	In which section is the information available? (Reagents and Tools Table, Materials and Methods, Figures, Data Availability Section)
**Plants:** provide species and strain, ecotype and cultivar where relevant, unique accession number if available, and source (including location for collected wild specimens).	Not Applicable	
**Microbes:** provide species and strain, unique accession number if available, and source.	Not Applicable	

Human research participants	Information included in the manuscript?	In which section is the information available? (Reagents and Tools Table, Materials and Methods, Figures, Data Availability Section)
If collected and within the bounds of privacy constraints report on age, sex and gender or ethnicity for all study participants.	Yes	Individuals affected with syndromic and non-syndromic optic neuropathy with negative or uncertain molecular diagnosis_mstxt & Table

Core facilities	Information included in the manuscript?	In which section is the information available? (Reagents and Tools Table, Materials and Methods, Figures, Data Availability Section)
If your work benefited from core facilities, was their service mentioned in the acknowledgments section?	Yes	Acknowledgements

Design

Study protocol	Information included in the manuscript?	In which section is the information available? (Reagents and Tools Table, Materials and Methods, Figures, Data Availability Section)
If study protocol has been **pre-registered, provide DOI in the manuscript**. For clinical trials, provide the trial registration number **OR** cite DOI.	Not Applicable	
Report the **clinical trial registration number** (at ClinicalTrials.gov or equivalent), where applicable.	Not Applicable	

Laboratory protocol	Information included in the manuscript?	In which section is the information available? (Reagents and Tools Table, Materials and Methods, Figures, Data Availability Section)
Provide DOI OR other citation details if **external detailed step-by-step protocols** are available.	Not Applicable	

Experimental study design and statistics	Information included in the manuscript?	In which section is the information available? (Reagents and Tools Table, Materials and Methods, Figures, Data Availability Section)
Include a statement about **sample size** estimate even if no statistical methods were used.	Yes	4 related individuals + 49 unrelated individuals_mstxt
Were any steps taken to minimize the effects of subjective bias when allocating animals/samples to treatment (e.g. **randomization procedure**)? If yes, have they been described?	Not Applicable	
Include a statement about **blinding** even if no blinding was done.	Not Applicable	
Describe **inclusion/exclusion criteria** if samples or animals were excluded from the analysis. Were the criteria pre-established?<br><br>If sample or data points were omitted from analysis, report if this was due to attrition or intentional exclusion and provide justification.	Yes	Negative panels-based molecular testing (unexplained cases)_mstxt &Table
For every figure, are **statistical tests** justified as appropriate? Do the data meet the assumptions of the tests (e.g., normal distribution)? Describe any methods used to assess it. Is there an estimate of variation within each group of data? Is the variance similar between the groups that are being statistically compared?	Not Applicable	

Sample definition and in-laboratory replication	Information included in the manuscript?	In which section is the information available? (Reagents and Tools Table, Materials and Methods, Figures, Data Availability Section)
In the figure legends: state number of times the experiment was **replicated** in laboratory.	Not Applicable	
In the figure legends: define whether data describe **technical or biological replicates**.	Not Applicable	

Ethics

Ethics	Information included in the manuscript?	In which section is the information available? (Reagents and Tools Table, Materials and Methods, Figures, Data Availability Section)
Studies involving **human participants**: State details of **authority granting ethics approval** (IRB or equivalent committee(s), provide reference number for approval.	Yes	Acknowledgements
Studies involving **human participants**: Include a statement confirming that **informed consent** was obtained from all subjects and that the experiments conformed to the principles set out in the WMA Declaration of Helsinki and the Department of Health and Human Services Belmont Report.	Yes	Acknowledgements
Studies involving **human participants:** For publication of **patient photos**, include a statement confirming that consent to publish was obtained.	Yes	Acknowledgements
Studies involving experimental **animals**: State details of **authority granting ethics approval** (IRB or equivalent committee(s), provide reference number for approval. Include a statement of compliance with ethical regulations.	Not Applicable	
Studies involving **specimen and field samples:** State if relevant **permits** obtained, provide details of authority approving study; if none were required, explain why.	Not Applicable	

Dual Use Research of Concern (DURC)	Information included in the manuscript?	In which section is the information available? (Reagents and Tools Table, Materials and Methods, Figures, Data Availability Section)
Could your study fall under dual use research restrictions? Please check biosecurity documents and list of **select agents and toxins** (CDC): https://www.selectagents.gov/sat/list.htm	Not Applicable	
If you used a select agent, is the security level of the lab appropriate and reported in the manuscript?	Not Applicable	
If a study is subject to dual use research of concern regulations, is the name of the **authority granting approval and reference number** for the regulatory approval provided in the manuscript?	Not Applicable	

Reporting

The MDAR framework recommends adoption of discipline-specific guidelines, established and endorsed through community initiatives. Journals have their own policy about requiring specific guidelines and recommendations to complement MDAR.

Adherence to community standards	Information included in the manuscript?	In which section is the information available? (Reagents and Tools Table, Materials and Methods, Figures, Data Availability Section)
State if relevant guidelines or checklists (e.g., **ICMJE, MIBBI, ARRIVE, PRISMA**) have been followed or provided.	Yes	ACMG variant classification guidelines_mstxt
For **tumor marker prognostic studies,** we recommend that you follow the **REMARK** reporting guidelines (see link list at top right). See author guidelines, under 'Reporting Guidelines'. Please confirm you have followed these guidelines.	Not Applicable	
For **phase II and III randomized controlled trials**, please refer to the **CONSORT** flow diagram (see link list at top right) and submit the CONSORT checklist (see link list at top right) with your submission. See author guidelines, under 'Reporting Guidelines'. Please confirm you have submitted this list.	Not Applicable	

Data Availability

Data availability	Information included in the manuscript?	In which section is the information available? (Reagents and Tools Table, Materials and Methods, Figures, Data Availability Section)
Have **primary datasets** been deposited according to the journal's guidelines (see 'Data Deposition' section) and the respective accession numbers provided in the Data Availability Section?	Not Applicable	
Were **human clinical and genomic datasets** deposited in a public access-controlled repository in accordance to ethical obligations to the patients and to the applicable consent agreement?	Yes	Accessibility of WES .bam files and CLINVAR references_Data Availability Section
Are **computational models** that are central and integral to a study available without restrictions in a machine-readable form? Were the relevant accession numbers or links provided?	Not Applicable	
If publicly available data were reused, provide the respective **data citations in the reference list**.	Not Applicable	
